# Improving the Accuracy of an R-CNN-Based Crack Identification System Using Different Preprocessing Algorithms

**DOI:** 10.3390/s22187089

**Published:** 2022-09-19

**Authors:** Mian Zhao, Peixin Shi, Xunqian Xu, Xiangyang Xu, Wei Liu, Hao Yang

**Affiliations:** 1 School of Rail Transportation, Soochow University, Suzhou 215006, China; 2 School of Transportation and Civil Engineering, Nantong University, Nantong 226019, China

**Keywords:** deep learning, crack detection, sparse feature, fast R-CNN algorithms, intelligent monitoring

## Abstract

The accurate intelligent identification and detection of road cracks is a key issue in road maintenance, and it has become popular to perform this task through the field of computer vision. In this paper, we proposed a deep learning-based crack detection method that initially uses the idea of image sparse representation and compressed sensing to preprocess the datasets. Only the pixels that represent the crack features remain, while most pixels of non-crack features are relatively sparse, which can significantly improve the accuracy and efficiency of crack identification. The proposed method achieved good results based on the limited datasets of crack images. Various algorithms were tested, namely, linear smooth, median filtering, Gaussian smooth, and grayscale threshold, where the optimal parameters of the various algorithms were analyzed and trained with faster regions with convolutional neural network features (faster R-CNN). The results of the experiments showed that the proposed method has good robustness, with higher detection efficiency in the presence of, for example, road markings, shallow cracks, multiple cracks, and blurring. The result shows that the improvement of mean average precision (mAP) can reach 5% compared with the original method.

## 1. Introduction

The computer vision-based detection of road cracks has a vital role in both life and engineering. Road cracks are often the initial manifestation of some diseases, and therefore, we need to detect the problem and take care of it as early as possible. In the past, the traditional manual inspection was time-consuming and laborious, the results were not always accurate, and it may have even posed a threat to the safety of the inspector’s life. In response, how to use computer vision to detect road defects in time and carry out maintenance as early as possible has become a popular topic. Deep learning has advanced at a rapid pace in recent years, and related algorithms continue to break through; many scholars have employed deep learning algorithms in road crack detection and achieved good outcomes. Zhang et al. were the first to propose the use of deep learning for road crack detection in 2016, which could directly use images taken by smartphones for manual annotation and automatically learn features [1]. The method they proposed greatly reduces the cost of detection, improves the disadvantages of manually produced features, and has better detection performance. However, the method has much room for improvement when it comes to detecting with speed and accuracy, and many scholars have subsequently continued to improve it. CrackNet is a new CNN-based pavement fracture detection system proposed by Zhang et al. [2] that is characterized by the absence of any pooling layers and that can reduce the output between layers. CrackNet has significantly better performance than the previous three-dimensional (3D) shadow modeling, but it still has many shortcomings, such as taking a long time to analyze and difficulty identifying cracks as tiny as a hair. Fei et al. were inspired by CrackNet and proposed CrackNet-V for pixel-level crack detection in pavements, which uses a deeper structure with fewer parameters to improve detection speed and accuracy [3]. Ye et al. put forward a deep learning-based crack detection method called Ci-Net, which is great improvement compared with edge detection [4]. Dung et al. suggested a crack semantic segmentation method based on full-depth convolution, choosing VGG16 as the FCN encoder backbone, and the semantic segmentation of this method could determine the crack path [5]. Although this method was able to capture the road crack path, further research is still needed to automatically quantify the crack size. Kalfarisi et al. proposed two crack detection and segmentation methods, faster region-based convolutional neural network forest edge detection (FRCNN-FED) and mask R-CNN, with detection results showing that speed is inversely proportional to performance; mask R-CNN had higher AP values but slower detection [6]. The two methods were applied to a unified framework to achieve crack segmentation, quantitative evaluation, and visualization. Huyan et al. proposed a pixel-level pavement crack detection method called CrackU-net that had good results for pixel-level pavement crack detection [7].

Although deep learning has produced excellent performance in intelligent crack detection, which no longer requires manual feature extraction and can now automatically learn features through the network, actual road crack pictures often have many complications. The well-known object detection algorithm faster R-CNN was tested [8], as seen in Section 3.3 in this paper, where the dataset shows road marking interference, shallow cracks, and blurred, multiple cracks. Other algorithms would be undetectable, so we needed to initially preprocess the dataset. Sparse representation and compressed sensing theory were applied to image processing.

Sparse representation has numerous uses in hyperspectral image anomaly object detection. Zhu et al. suggested both a target detection and a binary hypothesis model based on a sparse representation of object dictionary construction for hyperspectral image target detection, and the algorithms showed good performance [9]. Li et al. devised a method for detecting anomalies in hyperspectral images that accurately models the background using structural sparsity, and the results of the experiment show that the performance of this method is greatly optimized when compared with various current methods [10]. Ling et al. utilized a sparsity-based anomaly identification technique for hyperspectral images based on the fact that background pixels can be approximated and anomalous pixels estimated as sparse linear combinations of their neighborhoods [11]. Huyan et al. put forward a hyperspectral image anomaly detection method based on a dictionary of background and potential anomalies, where the anomalous part uses a sparse representation to model its properties [12]. In addition to the anomalous target detection of hyperspectral images, sparse representation is often used, for example, for video anomaly detection, fault diagnosis, and medical applications. Chu et al. combined sparse representation with unsupervised learning for video anomaly event detection, where sparse coding results for manual features generated from the input were used to guide unsupervised feature learning [13]. Sun et al. advanced a sparse representation framework based on a variational self-encoder latent space learning dictionary for anomaly detection that could reduce the dimensionality of high-dimensional data to save the cost of space [14]. Yuan et al. developed a dictionary learning algorithm to detect anomalous events in surveillance videos that explored new structural information in the sparse representation framework, and the algorithm confirmed its effectiveness with real data [15]. Sparse representation has also been used by several scholars in recent years in the field of intelligent transportation. Cheng et al. used sparse coding to identify the type of weather while driving, providing a key factor for tasks such as road detection [16]. Wang et al. proposed a method of identifying concrete cracks based on L2 sparse representation, and experiments showed that their method had high accuracy and efficiency [17]. Gao et al. performed a sparse representation of cab activities on spatially divided cells in cities that could perceive the spatiotemporal relationships between traffic flows and detect traffic anomalies in a timely manner [18].

Sparse representation has many applications in the field of anomaly detection. However, the essence of sparse representation is that in a sufficiently large training sample space, a class of objects can be roughly linearly represented by a subspace of similar samples in the training sample so that when the object has the entire sample space represented, the coefficients of its representation are sparse. Then, the model building requires that the sample space is large enough, the object is linear, and many other requirements, and the generalization of the method is poor. In contrast, the target detection method of deep learning requires less data and has high accuracy and generalization compared with the sparse representation method.

Compressed sensing theory was first proposed by Donoho [19], Candes and Tao [20], and Candes and Romberg [21]. The theory states that compressible signals can be sampled in a way that is far below the Nyquist standard and still recover the original signal accurately. The theory has been widely used in, for example, the fields of image processing, signal processing, medicine, and pattern recognition. Hu et al. proposed an intelligent fault diagnostic technique based on compressed sensing of improved multiple scale networks. Compressed sensing can decrease the amount of data and discover critical issue information while also providing enough training samples for subsequent learning [22]. Haneche et al. suggested a speech enhancement technique based on compressed sensing that could can subtract the effect of noise in speech, thereby achieving a more accurate recovery of speech [23]. Li et al. designed a method based on linear discriminant analysis and compressed sensors in order to identify the defective features on the surfaces of wood that could save time by reducing the complex training process using the principle of compressed sensing [24]. Zhang et al. suggested a wood disadvantage classification approach based on principal component analysis and compressed sensing, with the compressed sensing classifier offering fewer parameters, greater flexibility, faster calculation, and higher classification accuracy [25]. Böttger et al. utilized a compressed sensing-based method for monitoring and locating gray-scale texture defects in real time, which has significant advantages in terms of accuracy and speed [26]. Islam et al. proposed a deep learning framework based on compressed sensing to automatically detect pneumonia on images, and the method has strong robustness [27]. Shao et al. [28] proposed a feature learning and fault diagnosis of rolling bearings based on compressed sensing that could reduce the amount of complex data, exclude background noise, and improve diagnosis efficiency.

Although good results have been achieved using compressed sensing for anomaly detection, it is a difficult mathematical problem to find the appropriate sparse representation matrix, and the generalization of anomaly detection using compressed sensing theory is poor and the research and training process is not as easy as deep learning compared to target detection methods in the field of Faster R-CNN and other deep learning. But the idea of compressed sensing is still worthy of our reference.

Inspired by sparse representation and compressed sensing, four sparse feature algorithms were tested to highlight the cracks in the image and emphasize the features needed by denoising and enhancing the contrast between cracks and background. At the same time, data preprocessing methods can remove redundant information well without affecting the accuracy, reduce the data dimensionality and computation, and allow the model to learn the features needed better.

## 2. Method

**Figure 1:**
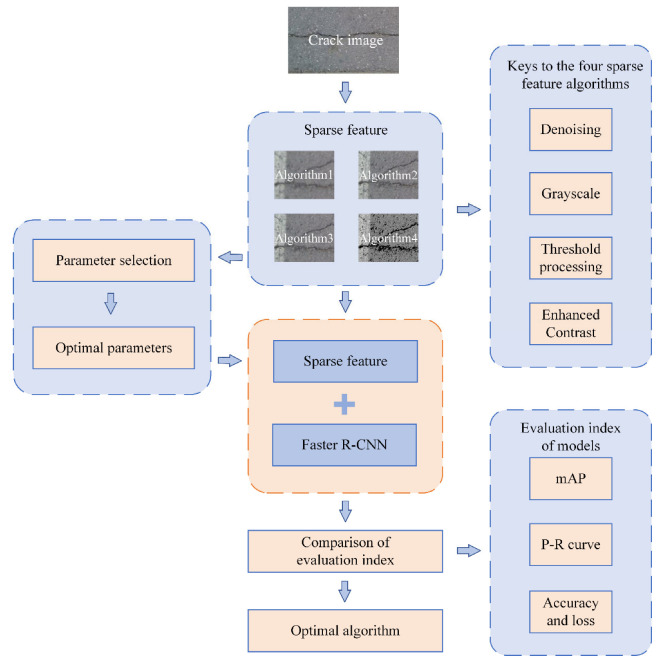
Pipeline of our method.

Figure 1 shows the pipeline of our method, first the dataset is preprocessed by four sparse feature methods, the keys of sparse feature are denoising, grayscale, thresholding, and contrast enhancement. Each sparse feature method selects multiple sets of parameters to compare to get the optimal parameters. Then the preprocessing dataset is trained using Faster R-CNN, and finally the optimal method is obtained by metrics comparison.

### 2.1. Data Preprocessing Methods

In the process of crack images acquisition, due to certain limitations in cost, electronic acquisition equipment, external environment, sensors, circuit structure, etc. may introduce noise, overexposure, over-darkness, blurring, etc. to make the crack not prominent enough, and the image transmission process will also be affected by these. The sparse representation and compressed sensing that inspired us can solve these problems, but they usually think from a mathematical point of view, with a complex computational process and poor automation. In contrast, our method combined with deep learning, which first processes the dataset with the idea of sparse features and then trains with Faster R-CNN, has better generalization, works on different datasets, and is faster and more accurate, too.

Data preprocessing is very significant in deep learning tasks. Firstly, we often encounter the problem of difficult data dimensions in real-life tasks, and if we can filter out some redundant features and keep important features, it will provide a good basis for subsequent learning. Secondly, after sparse feature, we can reduce the difficulty of learning, simplify the post-processing, while speeding up the training speed.

In this paper, we proposed a deep learning-based crack detection method, which initially uses the idea of image sparse representation and compressed sensing to preprocess the datasets. Only the pixels which represent the crack features remain, while most pixels of non-crack features are relatively sparse which can improve significantly the accuracy and efficiency of crack identification. The proposed method achieved good results based on the limited datasets of crack images. Various algorithms were tested namely linear smooth, median filtering, Gaussian smooth, and grayscale threshold, where the optimal parameters of various algorithms were analyzed, and trained with Faster R-CNN.

#### 2.1.1. Introduction of the 1st Algorithm

**Figure 2:**
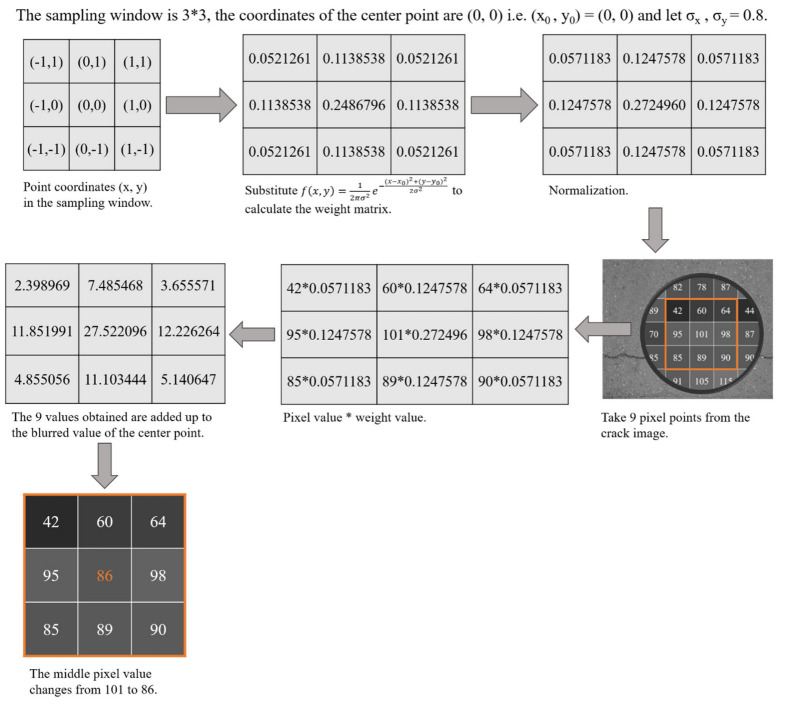
Pipeline of the 1st algorithm. Take the sampling window is 3 × 3, $\sigma_{x}$, $\sigma_{y}$ = 0.8, and the center point $\left(x_{0},y_{0}\right)$ is (0, 0) as an example.

Algorithm1 is a kind of linear smooth filter which can eliminate noise. It is very effective in suppressing noise obeying normal distribution, and is used extensively in image preprocessing. Algorithm1 is processed by convolving the image using a mask whose template coefficients obey a two-dimensional Gaussian distribution that drops as the distance from the template’s center grows. Algorithm1 may retain a lot of visual detail. The two-dimensional Gaussian function is shown in Equation (1) and the pipeline of algorithm1 is shown in Figure 2.

(1) $$f\left(x,y\right)=\frac{1}{2\pi \sigma^{2}}e^{-\left({\left(x-x_{0}\right)}^{2}+{\left(y-y_{0}\right)}^{2}\right)/2\sigma^{2}}$$

where $\frac{1}{2\pi \sigma^{2}}$ is a constant, $\left(x,y\right)$ is the coordinate of any point inside the mask, $\left(x_{0},y_{0}\right)$ is the coordinate of the center point of the mask, and σ is the standard deviation.

#### 2.1.2. Introduction of the 2nd Algorithm

**Figure 3:**
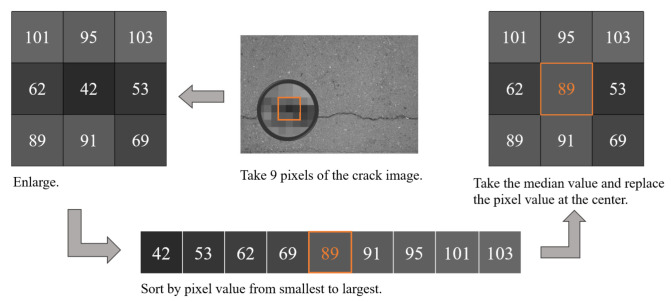
Pipeline of the 2nd algorithm.

Algorithm2 can remove the noise in the image, and at the same time can solve the problem of blurred image details generated in algorithm1, which is very effective for suppressing pretzel noise. Algorithm2 usually uses an odd number of sliding sampling windows, such as (2*n* + 1) × (2*n* + 1), *n* = 1,2,3……, and sorts the values in the window by the size of the pixel value of each pixel, then takes the median value after sorting and replaces it with the middle of the window. The pipeline of algorithm2 is shown in Figure 3. In Figure 3, the sampling window of 3 × 3 were taken as an example to illustrate the principle of algorithm2. First, the pixel values of these 9 pixels are sorted by size, and the middle value is 89, then the middle pixel of the sampling window is replaced with 89.

#### 2.1.3. Introduction of the 3rd Algorithm

Algorithm3 is a type of nonlinear smooth filter that is essentially Gaussian filter and is created to solve the edge blurring problem that occurs with algorithm1. The intensity of a pixel is replaced by a weighted average of the luminance values of the surrounding pixels in algorithm3, which takes into consideration not only the pixel’s Euclidean distance but also the radiation in the range domain of the pixel. The function of algorithm3 [29] is shown in Equations (2)–(5):

(2) $$I_{p}=\frac{1}{W_{p}}\underset{q\in s}{\sum }G_{\sigma_{s}}\left(∥p-q∥\right)G_{\sigma_{r}}\left(\left|I_{p}-I_{q}\right|\right)I_{q}$$

(3) $$W_{p}=\underset{q\in s}{\sum }G_{\sigma_{s}}\left(∥p-q∥\right)G_{\sigma_{r}}\left(\left|I_{p}-I_{q}\right|\right)$$

(4) $$G_{\sigma s}\left(∥p-q∥\right)=e^{-\frac{{\left(i-m\right)}^{2}+{\left(j-n\right)}^{2}}{2\sigma_{s}^{2}}}$$

(5) $$G_{\sigma r}\left(\left|I\left(p\right)-I\left(q\right)\right|\right)=e^{-\frac{\left[{\left(\left[i,j\right)-I\left(m,n\right)\right]}^{2}\right.}{2\sigma_{r}^{2}}}$$

where $I_{q}\;$ is the input image, $I_{p}\;$ is the filtered image, $G_{\sigma_{s}}$ is the spatial domain kernel, $G_{\sigma_{r}}$ is the image pixel domain kernel, $W_{p}\;$ is the sum of normalized weights, q is the input pixel point, m and n are the horizontal and vertical coordinates of the input pixel, p is the box center pixel point, and i and j are the coordinates of the box center pixel.

**Figure 4:**
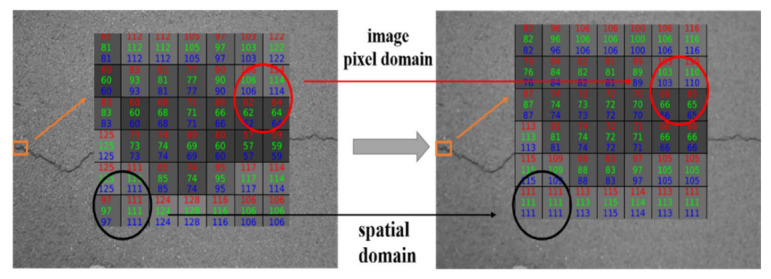
Schematic of the 3rd algorithm.

As shown in Figure 4, the crack intersects with the background mainly affected by the image pixel domain kernel, and the crack edge is preserved. And the background is mainly affected by the spatial domain kernel, and the pixels become smooth between them.

#### 2.1.4. Introduction of the 4th Algorithm

Algorithm4 is the process of adjusting the grayscale values of an image based on set values, eliminating pixels within the image that are above a certain value or below a certain value, which can give the entire image a distinct black and white effect. Algorithm4 enhances the subsequent work and facilitates the next step of model training, allowing the dataset to reduce unnecessary features, remove redundant information, and highlight the contours of objects of interest, such as removing backgrounds and highlighting crack features.

**Figure 5:**
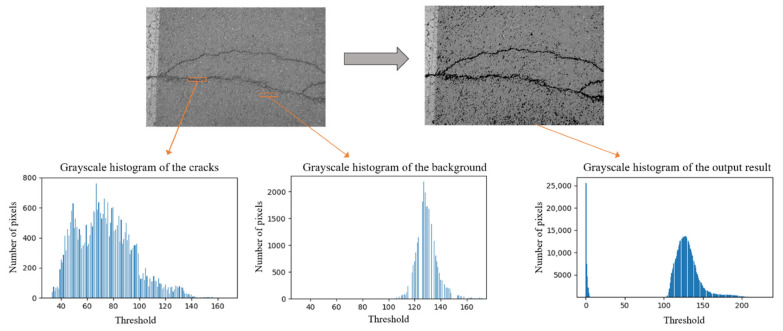
Schematic diagram of the 4th algorithm.

In this experiment, first grayscale the cracked image, intercept the crack and background parts separately and plot their grayscale histograms, then choose the threshold, which was used to divide the image into two parts, above and below the threshold, and give them different pixels. As shown in Equation (6) and Figure 5, *src*(*x*, *y*) indicates the pixel position of the original image, the part above the threshold is retained, and the part below or equal to the threshold takes the minimum value of zero.

(6) $$dst\left(x,y\right)=\left\{\begin{array}{cc} src\;\left(x,y\right) & if\;src\left(x,y\right)>t\\ 0 & \mathrm{otherwise} \end{array}\right.$$

### 2.2. Faster R-CNN

**Figure 6:**
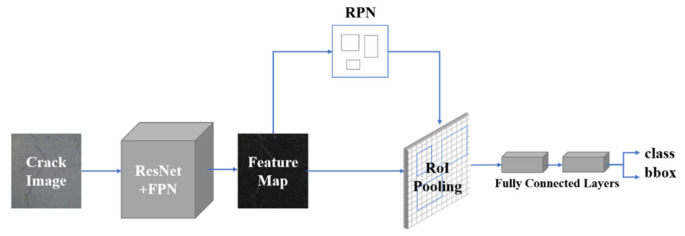
Pipeline of Faster R-CNN.

Faster R-CNN is divided into three critical sections: feature extraction, region proposal networks and region CNN as shown in Figure 6. We used Residual Network (ResNet) [30] and regional proposal network (FPN) [31] for the feature extraction part. After extracting the features, the feature extractor sends the five different size feature maps extracted to the following network.

#### 2.2.1. Network Structure of Faster R-CNN

Regarding the region proposal networks (RPNs) part, five feature maps are obtained from five identical RPNs and are employed in the creation of region proposals. Among them, the RPN creates anchors of various sizes to acquire a certain number of region proposal feature maps. Regarding the region CNN (R-CNN) part, the R-CNN takes the region suggestion feature maps obtained in the previous step with uniform size and then inputs them into the fully connected layer for classification and regression. Our network used ResNet101 as the backbone, which includes 5 classes of 100-layer convolution layers, and an average pooling layer for a total of 101 layers. We used batch normalization technology and we employed relu as the activation function. The entire Faster R-CNN network adopts a joint training strategy to sum each loss for unified gradient descent.

#### 2.2.2. Loss Function of Faster R-CNN

The loss function of Faster R-CNN includes Fast R-CNN loss [32] and RPN loss [8], which is multi-task, and both parts include classification loss $L_{cls}$ and regression loss $L_{reg}$, as shown in Equation (7):

(7) $$L\left(\left\{p_{i}\right\},\left\{t_{i}\right\}\right)=\frac{1}{N_{cls}}\underset{i}{\sum }L_{cls}\left(p_{i},p_{i}^{*}\right)\;+\lambda \frac{1}{N_{\mathrm{reg}\;}}\underset{i}{\sum }p_{i}^{*}L_{reg}\left(t_{i},t_{i}^{*}\right)$$

where i is the anchor index of the mini-batch, and $p_{i}$ is the likelihood of the anchor being the target. The ground truth label $p_{i}^{*}$ indicates whether the anchor is positive or negative; if the anchor is positive, $\left(p_{i}^{*}=1\right)$, and if the anchor is negative, $\left(p_{i}^{*}=0\right)$. $t_{i}=\left\{t_{x},t_{y},t_{w},t_{h}\right\}$ denotes the coordinate vector of the bounding box, and $t_{i}^{*}$ is the ground truth bounding box coordinate vector corresponding to the positive anchor. $N_{cls}$ is the mini-batch size and $N_{\mathrm{reg}\;}$ is the number of the anchor location, and they are normalized and weighted by a parameter λ. The classification loss $L_{cls}$ is the logarithmic loss for two classes, (object or non-object), and is shown in Equation (8); the regression loss as shown in Equation (9), where R is the $smooth_{L1}$ function, as shown in Equation (10).

(8) $$L_{cls}\left(p_{i},p_{i}^{*}\right)=-log\left[p_{i}^{*}p_{i}+\left(1-p_{i}^{*}\right)\left(1-p_{i}\right)\right]$$

(9) $$L_{reg}\left(t_{i},t_{i}^{*}\right)=R\left(t_{i}-t_{i}^{*}\right)$$

(10) $$smooth_{L1}\left(x\right)=\left\{\begin{array}{l} 0.5x^{2},\;\mathrm{if}\;\left|x\right|<1\\ \left|x\right|-0.5,\;\mathrm{otherwise}\; \end{array}\right.$$

#### 2.2.3. Mean of Average Precision (mAP)

The mAP is a key indicator of good or bad model performance in the field of object detection. The mAP value is the area enclosed under the precision recall curve after smoothing, as shown in Equation (13), where *P* is *Precision* and *r* is *Recall.*

(11) $$Precision\;=\frac{TP}{TP+FP}=\frac{TP}{all\;detections}$$

(12) $$Recall\;=\frac{TP}{TP+FN}=\frac{TP}{all\;ground\;truths}$$

(13) $$AP=\int_{0}^{1}P\left(r\right)dr$$

## 3. Analysis

Urban roads are maintained in time before cracks or cracks tend to appear, and future traffic volume is predicted to avoid overload when designing roads, so it is not an easy task to collect a large number of road crack pictures in a short time. Our method requires about 150 crack images to achieve good results. The dataset contains 150 representative crack images, of which 90% is the training data and 10% the testing data. We used a random flip method to expand the dataset by setting the probability of both horizontal and vertical flips to 0.5, which finally expanded the dataset by 50%.

### 3.1. Selection and Processing of Datasets

**Figure 7:**
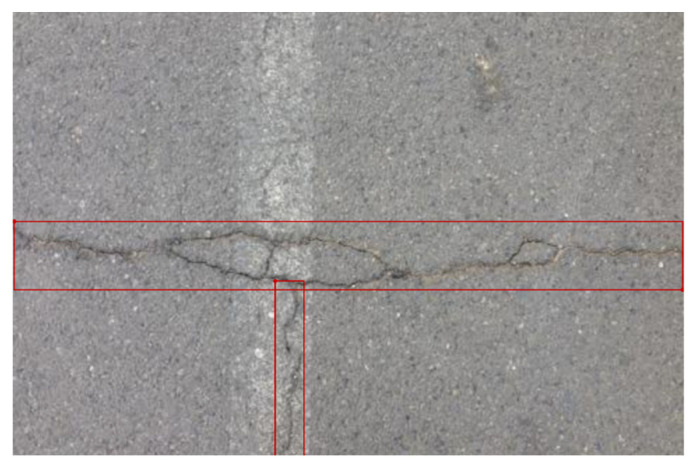
Labeling of cracks.

**Figure 8:**
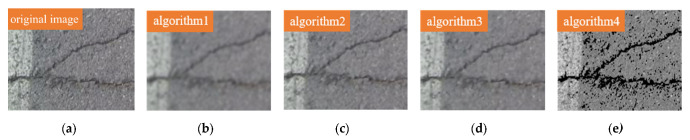
Original image and sparse feature image: (**a**) original image, (**b**) algorithm1, (**c**) algorithm2, (**d**) algorithm3, (**e**) algorithm4.

The dataset was partly derived from publicly available crack datasets on the web called CFD, and partly from actual road collection, taken by smartphones or cameras. The road crack dataset CFD in this study is publicly available (https://github.com/cuilimeng/CrackForest-dataset (accessed on 11 May 2017)). The crack images in the dataset are affected, for example, by broken road markings, shadows, water on the road, different road materials and blurring. The size of the image is 480 × 320 pixels, and all images were resized to 1333 × 800 pixels in our experiment. The optimal threshold will be influenced by the brightness, and the brightness of the images in the dataset will vary somewhat. However, in Figure 5, it can be seen that we determined the range of optimal thresholds based on statistical principles after counting the thresholds of the background and cracks in the dataset, and there will inevitably be individual images with poor results. At the same time, retaining more differences also allows the network to learn the features better for the actual detection and can improve the detection effect. If different datasets need to be used, the optimal threshold can be redetermined, but in general, the difference in brightness of the same dataset is not too large, so it is possible to separate the cracks from the background very well using our method. We marked the cracks in the images by drawing rectangular boxes, and the images marked are shown in Figure 7. The data are converted into the COCO format. The dataset after preprocessing by the four sparse feature methods introduced in Section 2.1 is shown in Figure 8.

Figure 7 shows the labeling of the dataset, and both the training data and testing data are labeled with rectangular boxes. If the crack feature in one image is shown in Figure 7 with multiple cracks in vertical and horizontal directions, multiple rectangular boxes are used to label it.

Figure 8 shows the comparison between the original image and the images after sparse feature, where (a) is the original image, and it can be seen that this image has the most detailed information and that there is much redundant information and noise; (b) is the image after algorithm1, and it can be seen that this image is the algorithm of removing the most redundant information, although the image has the disadvantage of blurred edges; (c) is the image after algorithm2, and it can be seen that this image removes part of the redundant information while retaining the crack information well; (d) is the image after algorithm3, in which it can be seen that this image is similar to algorithm2; and (e) is the image after algorithm4, which can be seen that the crack is clearly emphasized.

### 3.2. Experimental Details

Our experiments were conducted on the Ubuntu 20.04.3 operating system and NVIDIA GTX 3060 GPU and based on PyTorch 1.8.2 and CUDA 11.1. After several tests, the learning rate was set to a better effect of 0.005, and we trained a total of 24 epochs. We used a pretrained backbone based on ImageNet and executed a fine-tuning strategy to train our network. We also used SGD optimizer, batch normalization, and warm-up in training to improve the performance.

### 3.3. Crack Detection Results and Comparative Analysis

**Figure 9:**
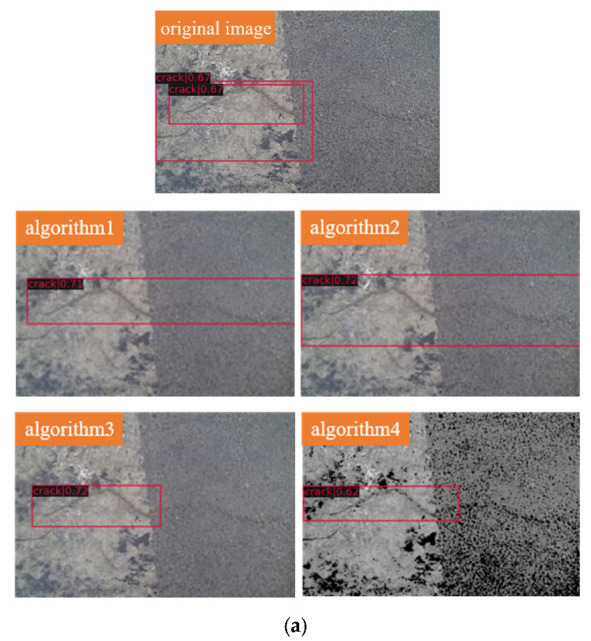

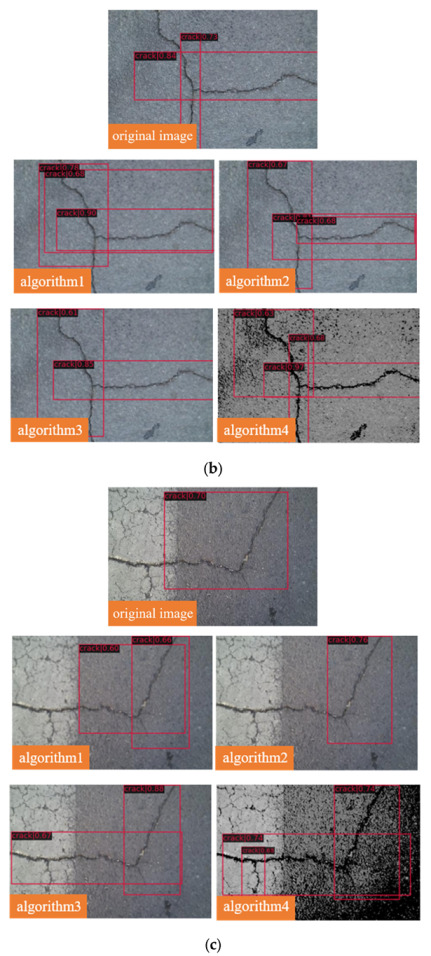

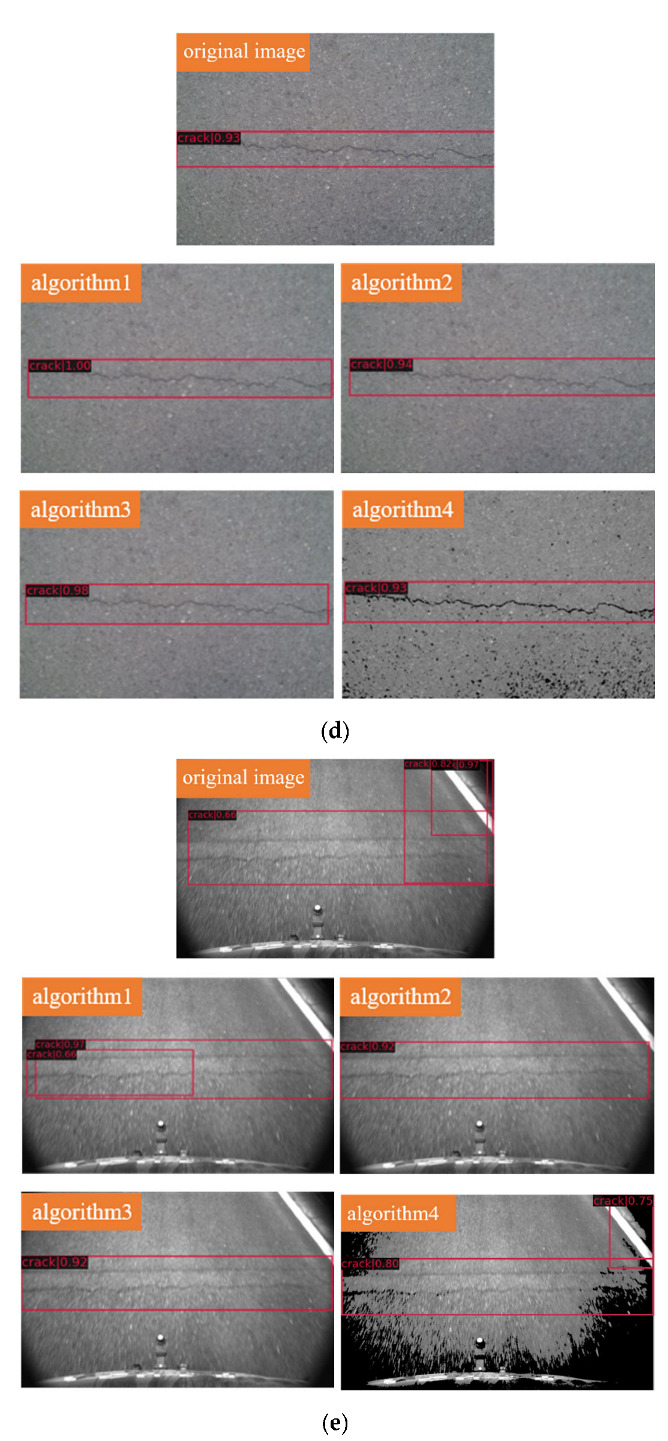

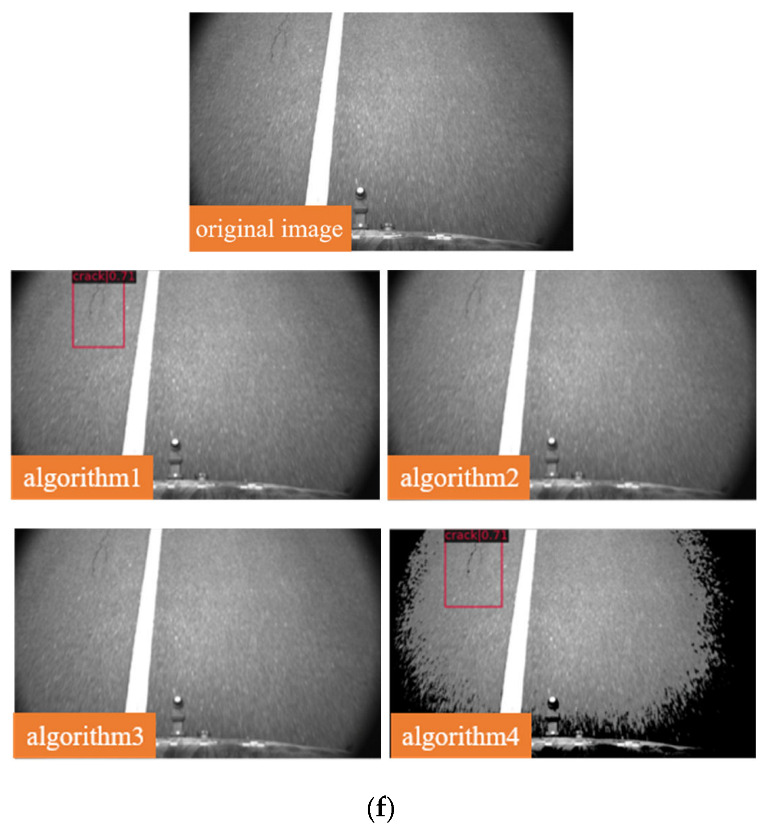
Testing results. (**a**) Results for testing data image1 with different methods. (**b**) Results for testing data image2 with different methods. (**c**) Results for testing data image3 with different methods. (**d**) Results for testing data image4 with different methods. (**e**) Results for testing data image5 with different methods. (**f**) Results for testing data image6 with different methods.

Figure 9 shows the detection results for the testing data. Each group is a comparison of the detection results for the same image after different sparse feature methods and unprocessed, algorithm1, algorithm2, algorithm3, and algorithm4 from front to back. All assays set the threshold to 0.6 or higher.

It can be clearly seen that for group (a), there is road marking interference in the picture, and the crack on the right side of the picture is thin and shallow; therefore, the unprocessed method cannot detect the whole crack well, while the whole crack can be detected completely after algorithm1 and algorithm2. Regarding the algorithm of both horizontal and vertical cracks in the same image, such as group (b), the unprocessed method cannot enclose the cracks completely, while the other four processed methods can detect the cracks completely, and the thresholds are all improved. Regarding the algorithm of group (c), where there are both horizontal and vertical cracks in the same image with the influence of road markings, the unprocessed method cannot enclose the cracks completely, while algorithm3 and algorithm4 can detect the cracks efficiently. Although for simple transverse cracks, such as in group (d), they all detect the cracks well, the thresholds are higher after the four sparse feature methods. The next groups (e) and (f) are both crack images that were collected with road condition acquisition equipment in cooperation with road inspection agencies, both of which are characterized, for example, by blurring and road marking line interference. The presence of detection errors, i.e., false positives, can be seen in the upper right corner of the untreated method in group (e), and there are no false positives at that position after algorithm1, algorithm2, and algorithm3; the threshold also increased substantially. The result after algorithm4 is still not satisfactory. The crack at the top left position in group (f) is prone to undetection, i.e., false negative, and algorithm1 and algorithm4 can avoid this situation and detect the crack. In summary, each sparse feature method is effective in improving the results of the assay.

## 4. Results

In this section, parameter selection, mAP, P–R curves, accuracy, and loss are compared and analyzed for five algorithms. First, several different sets of parameters are selected for the four algorithms for experimental comparison because the choice of parameters is related to the sparse feature results, which in turn affects the results of the model and can affect the key characteristics of the data if the parameters are not selected properly. Both mAP and P–R curves are measures of the results of the detection model, and the best algorithm can be clearly compared using these two indicators. Finally, accuracy and loss are two evaluation indicators of faster R-CNN, and in changes in accuracy and loss can be observed with in increases in iterations.

### 4.1. Parameter Selection of Four Sparse Feature Methods

In this section, multiple sets of parameters are set for experimental comparison and analysis for each algorithm. Algorithm1 chose the size of the sampling window ksize, the standard deviation of the sampling window kernel in the horizontal direction $\sigma_{x}$, and the standard deviation of the sampling window kernel in the vertical direction $\sigma_{y}$; algorithm2 chose the size of the sampling window ksize; algorithm3 chose the spatial distance d; algorithm4 chose the threshold value.

#### 4.1.1. Parameter Optimization and Comparative Analysis of the 1st Algorithm

**Figure 10:**
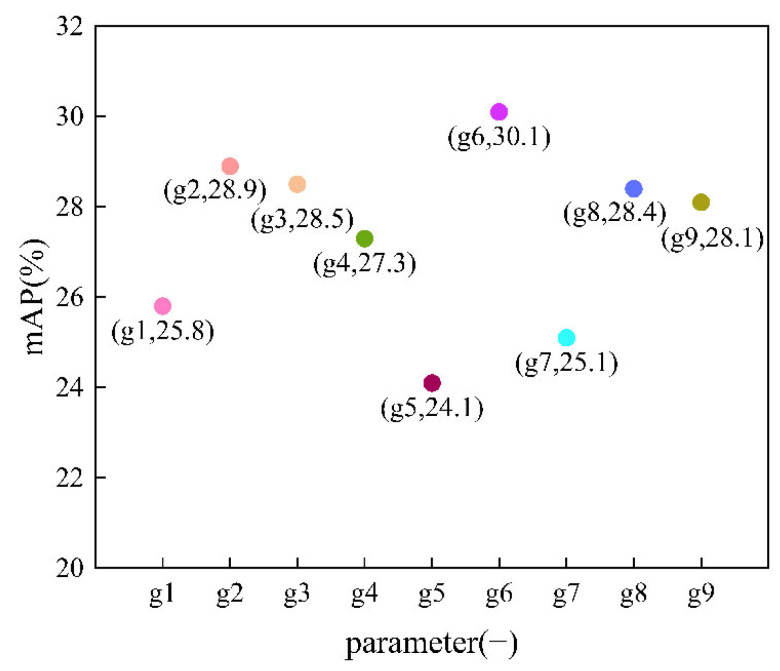
Variation of mAP of the model with parameter.

In algorithm1, there are three optional parameters, which are the size of the sampling window ksize, the standard deviation of the sampling window kernel in the horizontal direction $\sigma_{x}$, and the standard deviation of the sampling window kernel in the vertical direction $\sigma_{y}$, where ksize needs to be an odd number because these three parameters affect the sparse feature results, and if they are not selected properly, the sparse feature results will be worse; nine groups of parameters were chosen for testing as shown in Table 1. The comparison of the mAPs is shown in Figure 10.

In Figure 10, point (g1, 25.8) indicates that ksize is 3, the sampling window is 3 × 3, $\sigma_{x}$ is 0.8, $\sigma_{y}$ is 0.8, and the mAP of the model is 25.8; point (g2, 28.9) indicates that ksize is 5, the sampling window is 5 × 5, $\sigma_{x}$ is 0.8, $\sigma_{y}$ is 0.8, and the mAP of the model is 28.9; point (g3, 28.5) indicates that ksize is 5, the sampling window is 5 × 5, $\sigma_{x}$ is 1, $\sigma_{y}$ is 2, and the mAP of the model is 28.5; point (g4, 27.3) indicates that ksize is 5, the sampling window is 5 × 5, $\sigma_{x}$ is 3, $\sigma_{y}$ is 3, and the mAP of the model is 27.3; point (g5, 24.1) indicates that ksize is 5, the sampling window is 5 × 5, $\sigma_{x}$ is 10, $\sigma_{y}$ is 20, and the mAP of the model is 24.1; point (g6, 30.1) indicates that ksize is 7, the sampling window is 7 × 7, $\sigma_{x}$ is 0.8, $\sigma_{y}$ is 0.8, and the mAP of the model is 30.1; point (g7, 25.1) indicates that ksize is 7, the sampling window is 7 × 7, $\sigma_{x}$ is 1, $\sigma_{y}$ is 2, and the mAP of the model is 25.1; point (g8, 28.4) indicates that ksize is 7, the sampling window is 7 × 7, $\sigma_{x}$ is 3, $\sigma_{y}$ is 3, and the mAP of the model is 28.4; point (g9, 28.1) indicates that ksize is 9, the sampling window is 9 × 9, $\sigma_{x}$ is 0.8, $\sigma_{y}$ is 0.8, and the mAP of the model is 28.1.

The mAPs of the model with different parameters were compared, and the experiment results are shown in Figure 10. The horizontal coordinates in Figure 10 represent the parameter, and the vertical coordinates represent the mAP of the model. In Figure 10, it can be observed that when ksize is 7, i.e., the sampling window size is 7 × 7, mAP is the highest, and when $\sigma_{x}$ and $\sigma_{y}$ are 0.8, the result is better; the larger $\sigma_{x}$ and $\sigma_{y}$ are, the lower the mAP. The sparse feature results when ksize is 7, $\sigma_{x}$ is 0.8, and $\sigma_{y}$ is 0.8 are shown in Figure 8b, which shows that more redundant information is removed in this algorithm, but the edge information cannot be well preserved, in contrast with algorithm2. The results in Figure 9 show that the detection results after algorithm1 preprocessing greatly improved, and the results are more accurate, with less false detection and missed detection. In summary, the optimal parameters for algorithm1 are set as ksize of 7, sampling window of 7 × 7, $\sigma_{x}$ of 0.8, and $\sigma_{y}$ of 0.8, and the highest mAP of 30.1 is achieved with this parameter.

#### 4.1.2. Parameter Optimization and Comparative Analysis of the 2nd Algorithm

**Figure 11:**
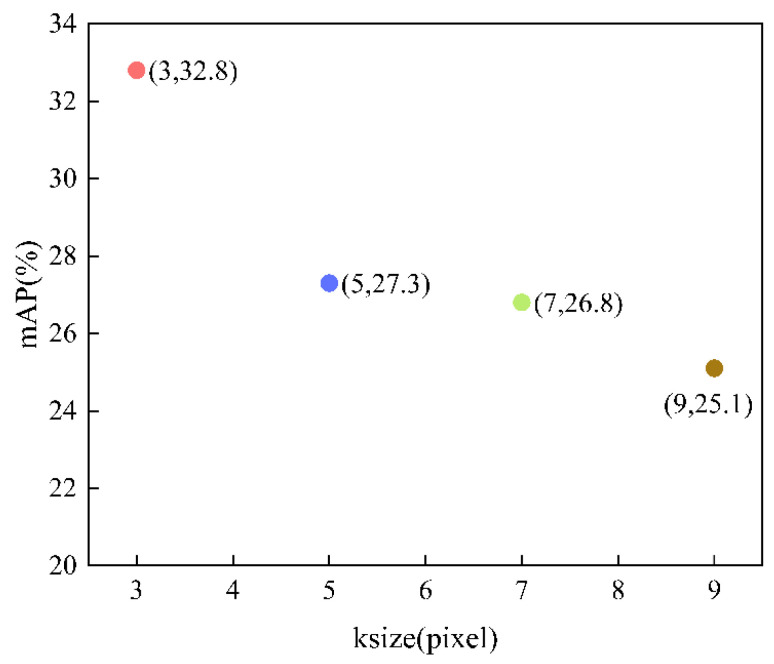
Variation of mAP of the model with ksize.

In algorithm2, an optional parameter is the size of the sampling window ksize, which must be an odd number greater than 1 and equal in length and width. Because the size of the sampling window affects the sparse feature results, if the sampling window is too small, the preprocessing will not change much, whereas if the sampling window is too large, the image will become blurred; therefore, different sampling window sizes were chosen for testing, and the best parameters were compared and analyzed. Four different ksize were chosen, 3, 5, 7, and 9, making the sampling windows 3 × 3, 5 × 5, 7 × 7, and 9 × 9. The comparison of the mAPs is shown in Figure 11.

In Figure 11, point (3,32.8) indicates that ksize is 3, the sampling window is 3 × 3, and mAP of the model is 32.8; point (5,27,3) indicates that ksize is 5, the sampling window is 5 × 5, and the mAP of the model is 27.3; point (7,26.8) indicates that ksize is 7, the sampling window is 7 × 7, and the mAP of the model is 26.8; point (9.25.1) indicates that ksize is 9, the sampling window is 9 × 9, and the mAP of the model is 25.1.

The mAPs of the models with different ksizes were compared, and the experiment results are shown in Figure 11. The horizontal coordinates in Figure 11 represent the parameter ksize, and the vertical coordinates represent the mAP of the model. The higher the mAP, the better the model. It can be clearly seen that the mAP is highest when the ksize is 3 × 3, reaching 32.8, while the larger the sampling window, the lower the mAP. Algorithm2 can protect the edge information of the image well under certain conditions, but choosing a too-large sampling window will make the image blurred and lose too much feature information. When the sampling window is 3 × 3, algorithm2 can remove the redundant information while retaining the key crack feature information to the maximum extent, as shown in Figure 8c, whereas when the sampling window is increased to 9 × 9, the image is already so blurred that too much key information is lost. As shown in Figure 9, when the sampling window of algorithm2 is 3 × 3, there is more accurate detection in the testing data compared with the unprocessed algorithm. Finally, we set the optimal parameter for algorithm2 as ksize of 3 and mAP of 32.8.

#### 4.1.3. Parameter Optimization and Comparative Analysis of the 3rd Algorithm

**Figure 12:**
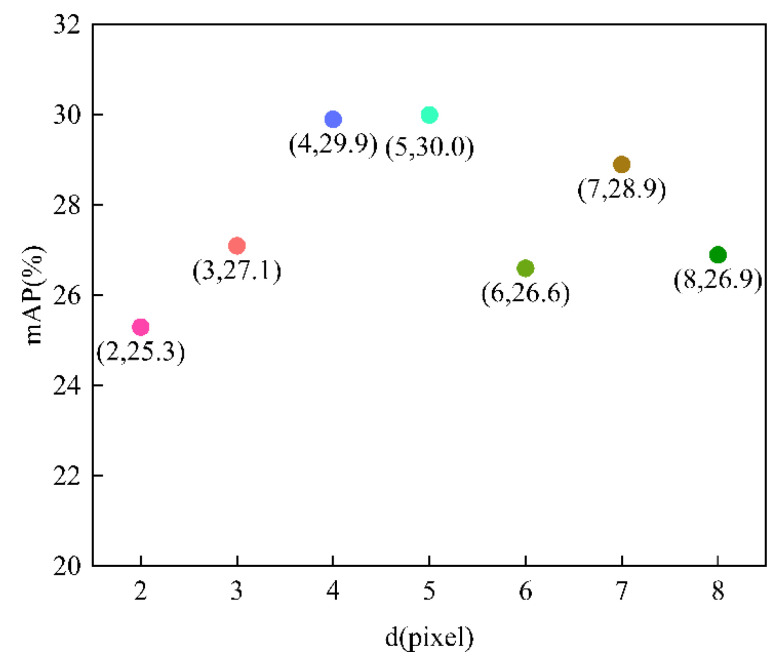
Variation of mAP of the model with d.

In algorithm3, the optional parameter is the spatial distance d, which is the diameter of the current pixel centroid, because the size of the window should cover the size of the noise to be removed: If the window is too small, the denoising effect is not obvious, and if the window is too large, it will be over denoised, so it is important to choose the right parameter. Here, seven different parameters d are chosen, namely, 2, 3, 4, 5, 6, 7, and 8. The comparison of the mAPs is shown in Figure 12.

In Figure 12, point (2, 25.3) indicates that d is 2, and the mAP of the model is 25.3; point (3, 27.1) indicates that d is 3, and the mAP of the model is 27.1; point (4, 29.9) indicates that d is 4, and the mAP of the model is 29.9; point (5, 30.0) indicates that d is 5, and the mAP of the model is 30.0; point (6, 26.6) indicates that d is 6, and the mAP of the model is 26.6; point (7, 28.9) indicates that d is 7, and the mAP of the model is 28.9; point (8, 26.9) indicates that d is 8, and the mAP of the model is 26.9.

The mAPs of the model with different d were compared, and the experiment results are shown in Figure 12. The horizontal coordinates in Figure 12 represent ksize, and the vertical coordinates represent the mAP of the model. According to Figure 12, as d increases, mAP first increases and then decreases, where mAP is close to and also the highest point when d is 4 and 5, and the mAP reaches 30.0 when d is 5. Algorithm3 can solve the problem of blurred edges appearing in algorithm1, as shown in Figure 8d; the crack features can be well preserved, and the redundant information is removed at the same time. As shown in Figure 9, when the d of algorithm3 is 5, there is a more accurate detection result in the testing data compared with the unprocessed algorithm. In summary, the optimal parameter setting for algorithm3 is d equal to 5.

#### 4.1.4. Parameter Optimization and Comparative Analysis of the Fourth Algorithm

**Figure 13:**
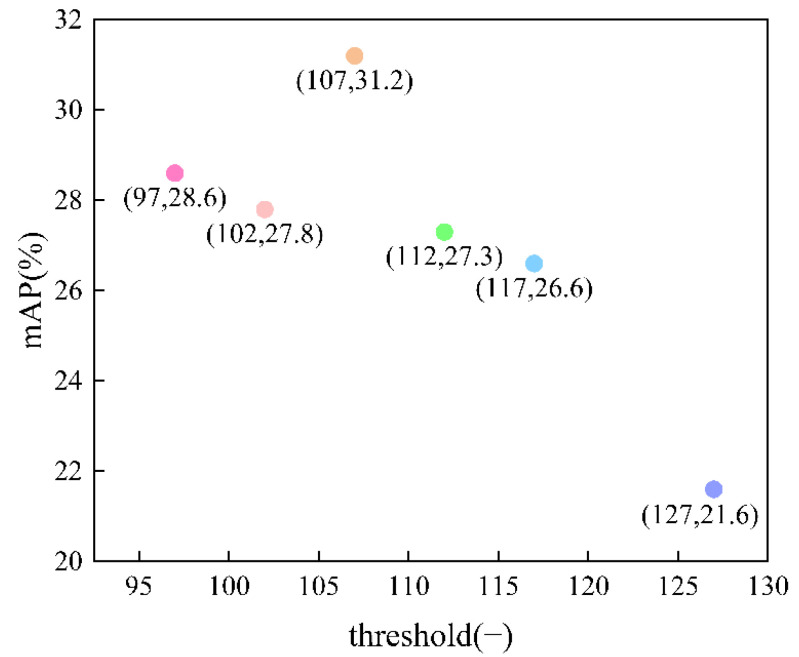
Variation of mAP of the model with threshold.

In algorithm4, the optional parameter is the threshold: Pixels larger than the set threshold are retained, and pixels smaller than this threshold become 0. If the threshold is set too large, part of the background pixels and the cracked part become 0, making it difficult to distinguish the background from the crack, and if the threshold is set too small, the preprocessing is not obvious, and even some shallow cracks are larger than the set threshold and are not emphasized. Six thresholds were set: 97, 102, 107, 112, 117, 127. The comparison of the mAPs is shown in Figure 13.

In Figure 13, the point (97, 28.6) indicates that threshold is 97, and the mAP of the model is 28.6; point (102, 27.8) indicates that threshold is 102, and the mAP of the model is 27.8; point (107, 31.2) indicates that threshold is 107, and the mAP of the model is 31.2; point (112, 27.3) indicates that threshold is 112, and the mAP of the model is 27.3; point (117, 26.6) indicates that threshold is 117, and the mAP of the model is 26.6; point (127, 21.6) indicates that threshold is 127, and the mAP of the model is 21.6.

The mAPs of the model with threshold were compared, and the experiment results are shown in Figure 13. The horizontal coordinates in Figure 13 represent the threshold, and the vertical coordinates represent the mAP of the model. Based on experience, the threshold for images is generally set to 127. We first experimented with the threshold of 127, but the mAP obtained was 21.6. After observing the dataset, we concluded that the reason was that the cracks were not prominent enough and not well separated from the background due to setting the threshold too high. Therefore, the threshold was lowered; the range was set between 102 and 117, and the dataset performed better with the cracks more prominent. The method did not differ much from the original dataset after preprocessing when the threshold was set too small and lost too much feature information or even failed to observe the cracks when the threshold was set too large. From the comparison of the results in Figure 13, it is clear that the model has the highest mAP and the best results when the threshold is set to 107. In summary, the optimal parameter for algorithm4 is threshold set to 5.

### 4.2. Comparison and Analysis of mAP

In this section, the results of the unprocessed algorithm and the four sparse feature algorithms are compared, where the best parameters obtained from the comparative analysis in Section 4.1 are selected for all four sparse feature algorithms. The metric used to perform the comparison is mAP, introduced in Section 2.2.3; mAP is a valid metric for target detection accuracy. The results are shown in Table 2, where mAP is the average AP at 0.05 intersection over union (IOU) intervals from IOU = 0.5 to IOU = 0.95: mAP50 is the AP at IOU = 0.5, and mAP75 is the AP at IOU = 0.75.

Table 2 shows the mAP, mAP50, and mAP75 for the unprocessed algorithm, the four sparse feature algorithms, and the combined sparse feature algorithm. The bold text represents the method with the best results. The four sparse feature algorithms were experimentally demonstrated to have better detection results compared with the unprocessed algorithm, but the effect became worse after combining the sparse feature algorithms.

Faster R-CNN + algorithm2 has the most obvious improvement, with the mAP increasing from the original 27.8 to 32.8, followed by faster R-CNN + algorithm4, with the mAP increasing to 31.2. mAP50 with faster R-CNN + algorithm1 and with faster R-CNN + algorithm2 improve very significantly to 69.5 and 69.0, respectively. mAP75 with faster R-CNN + algorithm4 improves from the original 18.4 to 26.4. As shown in Figure 7, the redundant information of some images is removed after preprocessing, and in particular, Figure 8c retains the crack features well while removing the redundant information. As seen in Figure 9, the crack images in the testing data have more accurate detection results after preprocessing. We randomly selected two combinations for testing, algorithm2 + algorithm4 and algorithm1 + algorithm2 + algorithm3 + algorithm4, and their mAPs were, respectively, 24.8 and 9.3; we found that these methods were less effective when stacked than when they were when used alone. Based on the results of the experiments, we can conclude that sparse feature is very effective in improving crack detection models.

### 4.3. Comparison and Analysis of Precision–Recall Curve

**Figure 14:**
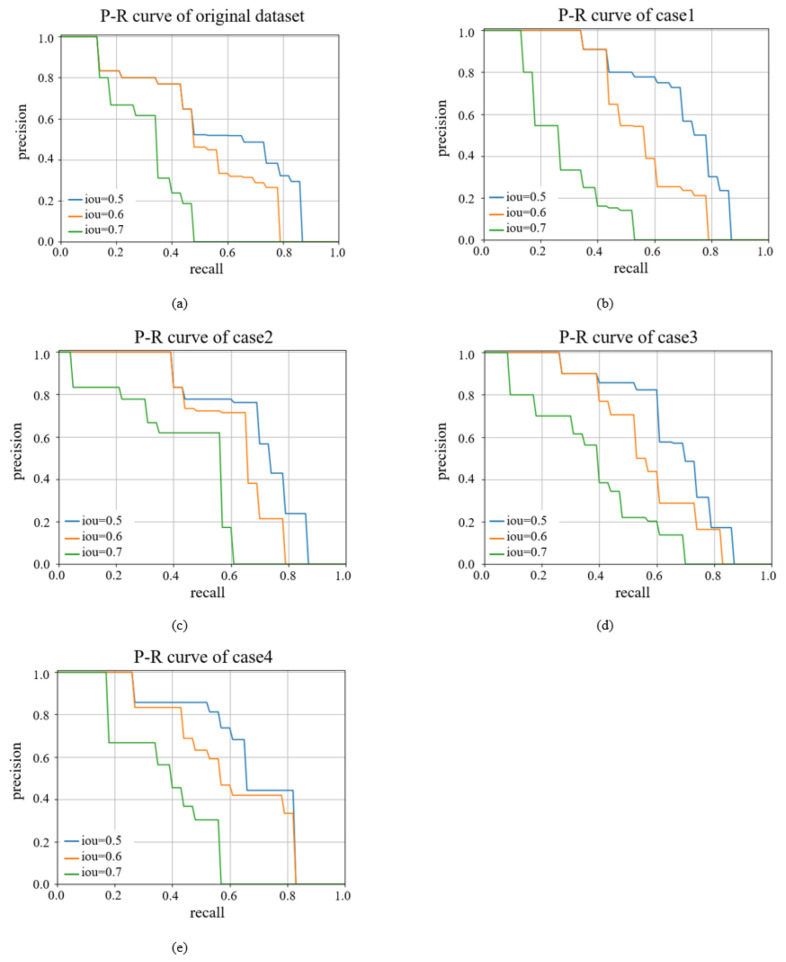
Precision–recall curve in bounding boxes using different methods. (**a**) Faster R-CNN, (**b**) Faster R-CNN +algorithm1, (**c**) Faster R-CNN + algorithm2, (**d**) Faster R-CNN + algorithm3, (**e**) Faster R-CNN + algorithm4.

In this section, the comparison of P–R curves of the models trained with the unprocessed dataset and the dataset after four sparse feature algorithms is shown. The P–R curve is the variation in the accuracy and completeness with IoU. IoU, intersection over union, calculates the intersection rate between the predicted border and the real border. Three representative IoUs were selected to show their P–R curves. The P–R curve intersects the x-axis and y-axis to enclose an area, and the larger the area, the better the performance of the model. The results of the P–R curves are shown in Figure 14.

The P–R curves of the models trained with the unprocessed dataset and the dataset after four sparse feature algorithms are compared, and the results of the P–R curves are shown in Figure 14. The horizontal coordinates in Figure 14 represent recall, and the vertical coordinates represent precision. The blue line represents the P–R curve when IoU is 0.5, the orange line represents the P–R curve when IoU is 0.6, and the green line represents the P–R curve when IoU is 0.7. The precision–recall curves of the four methods are shown as bounding boxes in Figure 14. It can be obviously seen that the effect of the four methods after sparse feature are significantly improved: When IoU is 0.5, the best results are obtained after algorithm2 preprocessing; when IoU is 0.6, the best results are obtained after algorithm2 preprocessing; and when IoU is 0.7, the best result is obtained after algorithm4 preprocessing.

### 4.4. Accuracy and Loss of Faster R-CNN

**Figure 15:**
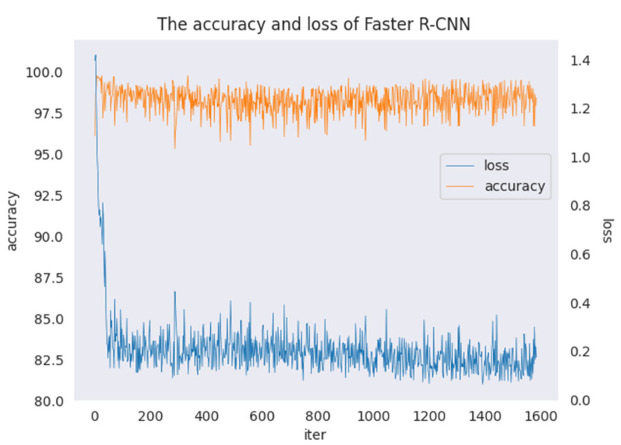
The accuracy and loss of faster R-CNN.

Observing the accuracy and loss during the training of Faster R-CNN, two key indicators evaluate how well the model is trained. Accuracy should gradually increase and stabilize, and loss should gradually decrease and stabilize—otherwise, the model has a problem at some step. Since the untreated method is similar to the accuracy and loss plots of the four algorithms, only the accuracy and loss of the untreated algorithm are shown here. The accuracy and loss of faster R-CNN are presented in Figure 15.

The variations in the accuracy and loss with faster R-CNN according to the number of iterations is shown in Figure 15, where the horizontal coordinates represent the number of iterations, the left vertical coordinates represent the accuracy, the right vertical coordinates represent the loss, the orange dash represents accuracy, and the blue dash represents loss. In Figure 15, it can be seen that the accuracy of faster R-CNN finally stabilized at 98, and the initial loss with faster R-CNN is 1.4, which first decreases rapidly with increases in the number of iterations, then decreases steadily, and finally stabilizes at about 0.2. The variations in accuracy and loss show that the model is trained without any anomaly and the results are good.

## 5. Conclusions

The appearance of cracks is an early warning of road diseases, which will cause more serious traffic problems if not treated in time; therefore, it is especially important to detect cracks in a timely and efficient manner. In this paper, the faster R-CNN algorithm was adopted and improved, which is an efficient and high-accuracy method of detecting cracks in a timely manner.

(i) An intelligent crack detection method based on deep learning algorithm is investigated to improve the accuracy and reliability of small sample sets.
(ii) Combined with sparse representation and compressed sensing, the dataset was preprocessed, and various preprocessing algorithms were compared.
(iii) The optimal parameters for different algorithms were compared and analyzed, and the corresponding mAP improved significantly. Among them, the mAP of algorithm2 is the largest, reaching 5%.
(iv) The results show that the crack detection effects in complex situations such as road marking interference, shallow cracks multiple cracks, and ambiguity were significantly improved.
(v) Algorithm1 is aimed at shallow cracks, with better results for road marking interference, lateral cracks, blurred pictures, and small-area cracks; algorithm2 is aimed at shallow cracks, with better results for road marking interference, multiple cracks, and blurred pictures have better results. With Algorithm3 for multiple cracks, there are better results for road marking interference, lateral cracks, and blurred pictures. Algorithm4 is aimed at multiple cracks, and there are better results for road marking interference and blurred pictures.

For further work, we will study how to make better feature selection, remove redundant features to the greatest extent, retain the most real cracks, allow the model to be better trained, and obtain better detection results. In order to further improve the effects, we will continue to study other feature sparse methods and try more dimensional sparse feature methods, such as color saturation and wavelet. Our research is preliminary, and further research is needed on how to identify the types of damage because different repair methods may be used for different types of damage (cracking) as well as how to classify detected cracks as harmless or repair them immediately. Since the focus of this research paper is on the improvement of detection effects after adding sparse feature processing before the deep learning method, our dataset only selected from images of longitudinal, transverse, and bifurcation cracks in asphalt pavement. Future refinement of the dataset is needed to include other types of damage that are significant and require precise automatic detection in the road engineering: block, fatigue, edge, and reflection cracks, and moreover potholes as well as losses of binder and aggregate in the road surface.

## Figures and Tables

**Table 1:** Parameter optimization of the first algorithm.

g	Ksize	$\sigma_{x}$	$\sigma_{y}$
g1	3	0.8	0.8
g2	5	0.8	0.8
g3	5	1	2
g4	5	3	3
g5	5	10	20
g6	7	0.8	0.8
g7	7	1	2
g8	7	3	3
g9	9	0.8	0.8

**Table 2:** Comparison of mAPs for different algorithms.

	mAP (%)	mAP50 (%)	mAP75 (%)
Faster R-CNN	27.8	57.6	18.4
Faster R-CNN + algorithm1	30.1	**69.5**	24.6
Faster R-CNN + algorithm2	**32.8**	69.0	23.7
Faster R-CNN + algorithm3	30.0	65.8	19.1
Faster R-CNN + algorithm4	31.2	65.7	**26.4**
Faster R-CNN + algorithm2 + algorithm4	24.8	59.1	18.2
Faster R-CNN + algorithm1 + algorithm2 + algorithm3 + algorithm4	9.3	36.6	1.2

## Data Availability

The data presented in this study are available on request from the corresponding author.
